# Simple spectrophotometric methods for the quantitative analysis of two binary mixtures containing paracetamol as a major component

**DOI:** 10.1186/s13065-025-01643-7

**Published:** 2025-10-27

**Authors:** Karin M. Guirguis, May M. Zeid, Rasha A. Shaalan, Tarek S. Belal

**Affiliations:** 1 Pharmacy Program, Kazan Federal University, Cairo Branch, Cairo, Egypt; 2 Pharmaceutical Chemistry Department, Faculty of Pharmacy, Pharos University in Alexandria, Canal El-Mahmoudia Street, Beside Green Plaza Complex, Alexandria, 21648 Egypt; 3 Pharmaceutical Analytical Chemistry Department, Faculty of Pharmacy, University of Alexandria, Elmessalah, Alexandria, 21521 Egypt

**Keywords:** Paracetamol, Meloxicam, Domperidone, Direct zero-order, First-order derivative, Ratio difference method, Greenness assessment

## Abstract

This work demonstrates simple and reliable spectrophotometric methods for the simultaneous determination of paracetamol (PAR) and meloxicam (MEL) in mixture I as well as PAR and domperidone (DOM) in mixture II in bulk form and laboratory-made tablets. Successful determination of mixture I was accomplished using direct zero-order spectrophotometry at 361 nm for MEL and first-order derivative (^1^D) spectrophotometry by measuring the peak at 342 nm and the trough at 262 nm for MEL and PAR respectively. On the other hand, a ratio difference method was suggested for the analysis of mixture II. The difference between the ratio spectra amplitudes at 256 and 288 nm were recorded for PAR determination, and 216 and 288 nm for DOM quantitation using 50 µg/mL DOM and PAR respectively as divisors. The efficacy of the proposed procedures was assessed using ICH criteria for linearity, ranges, precision, accuracy, detection, and quantitation limits. With regard to mixture I, the calibration curves showed linearity in the ranges of 3–30 µg/mL for MEL (zero-order method) and 2.5–30 and 3–15 µg/mL for MEL and PAR, respectively (first-order method), with correlation values of at least 0.9991.Whereas for mixture II, with correlation coefficients of 0.9999, the calibration curves for PAR and DOM were linear in the ranges of 3–70 and 2.5–15 µg/mL, respectively.The established procedures were used to analyze the combinations in the lab-prepared pills, and assay results were compared with reported methods. Greenness of the devised spectrophotometric procedures was assessed using the Analytical Eco-Scale and the Analytical Greenness metric (AGREE).

**Supplementary Information:**

The online version contains supplementary material available at 10.1186/s13065-025-01643-7.

## Introduction

**Fig. 1:**
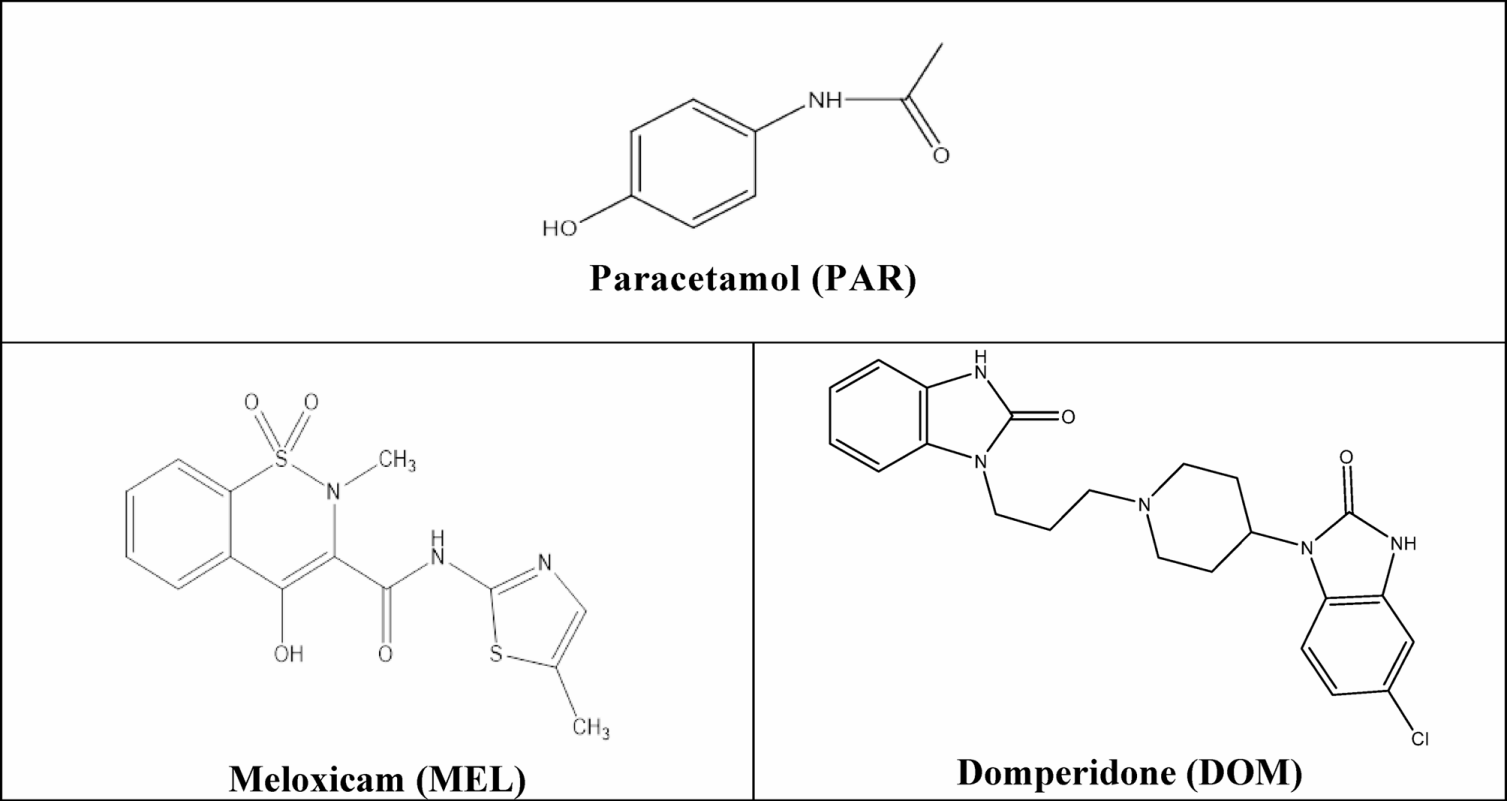
Chemical structures of the studied drugs

Paracetamol (PAR), a para-aminophenol derivative, has antipyretic, analgesic activities and modest anti-inflammatory effect. Paracetamol is found in several pharmaceutical binary mixtures with other drugs including meloxicam (MEL) and domperidone (DOM) (Fig. 1).

The combination of PAR and MEL is used as a painkiller and to treat inflammation in diseases such as osteoarthritis, rheumatoid arthritis, and ankylosing spondylitis. Additionally, it is used to treat toothaches, ear and throat discomfort, back pain, and muscle pain [1]. PAR and MEL binary mixture was simultaneously determined in several analytical reports. They proposed different spectrophotometric methods such as simultaneous equations [2, 3]. , absorbance ratio [2, 3] and absorbance correction method [2]. In addition, some chromatographic methods, such as high performance liquid chromatography (HPLC) [1, 4, 5] and high performance thin layer chromatography (HPTLC) [6] were recommended. The examination of this mixture encountered two significant problems; first, the spectral overlapping of both drugs was a major problem. The second problem was the presence of MEL as a minor component in the mixture where the tablets dosage form comprised PAR and MEL in a ratio of 130:3 [1]. The proposed spectrophotometric method succeeded to determine MEL using direct zero-order spectrophotometry at 361 nm and first-order derivative (^1^D) spectrophotometry by measuring the peak at 342 nm in methanol. Moreover, the proposed first-order derivative method could also determine PAR by measuring the trough at 262 nm.

A tablet dosage form containing both PAR and DOM is also commercially available in the international market. This combination is used as anti-emetic and to relief pain associated with gastrointestinal (GIT) disorders as well as for treatment of migraine. Several analytical methods were presented for the simultaneous determination of PAR and DOM binary mixture. Spectrophotometry was one of the techniques applied using first order derivative measurement [7] and the famous simultaneous equations, also known as Vierordt’s method [8]. Additionally, the binary mixture was assayed using high performance liquid chromatography with diode array detection (HPLC-DAD) [9], voltammetry [10] and HPTLC [11, 12]. Similar to the previous mixture (PAR/MEL), there are two major problems facing the analysis of PAR/DOM mixture. Firstly, the spectral overlap of both drugs which hinders the direct spectrophotometric analysis. Secondly, DOM is considered the minor component where the pharmaceutical combination PAR/DOM is present in a ratio of 25:1 [8, 10]. For this binary mixture, ratio difference method was suggested for resolution of the overlapped PAR/DOM spectra. The proposed techniques were thoroughly verified and evaluated for environmental friendliness utilizing newly released greenness evaluation tools.

## Experimental

### Instrumentation

All spectrophotometric measurements were carried out using a Thermo Spectronic Helios Alpha double-beam UV-visible spectrophotometer (Thermo Electron Corporation, Waltham, MA, USA) linked to Harvest computer system. The measurements were taken in 1-cm quartz cells. The Harvest computer system is linked to Panasonic impact dot matrix printer KX-P3626.

### Materials and reagents

Paracetamol (PAR), meloxicam (MEL), domperidone (DOM) and pill fillers (maize starch, microcrystalline cellulose “Avicel”, magnesium stearate, colloidal silica “Aerosil”) were kindly provided by Pharco Pharmaceuticals Company, Alexandria, Egypt. HPLC-grade methanol (Fisher Scientific, UK) and dimethylformamide (DMF) (Loba chemie, India) were utilized.

### General procedures and construction of calibration graphs

#### Mixture I (PAR and MEL)

##### Preparation of stock and working solutions

A 1000 µg/mL PAR standard solution was made using methanol. MEL standard solution was freshly prepared by first dissolving the drug substance in minimal amount (not more than 1 mL) of DMF, then diluting to a volume of 10 mL with methanol to reach a concentration of 1000 µg/mL. The stock solution was then wrapped with aluminum foil to protect it from light. By accurately transferring the volumes of the previously made standard solutions of PAR and MEL into two different sets of 10-mL volumetric flasks, the working solutions were created. Dilution was made to volume with methanol to get final concentration ranges of 3–15 µg/mL for PAR and 3–30 µg/mL for MEL. The zero order absorption spectra of the prepared working solutions were scanned from 200 to 400 nm against solvent blank and saved to the computer.

##### Zero order spectrophotometric method for determination of MEL

The stored spectra of MEL were used directly for construction of calibration curve by plotting the peak amplitude at λ_max_ 361 nm versus the corresponding concentrations, and then the regression equation was computed.

##### First- order derivative spectrophotometric method (^1^D)

For determination of PAR, the first derivative (^1^D) spectra (obtained by the spectrophotometer’s software) were recorded. The values of the ^1^D amplitude were measured from zero to trough at 262 nm. Similarly, for determination of MEL, the ^1^D amplitudes were measured from zero to peak at 342 nm. The calibration graphs were constructed by plotting the collected ^1^D measurements against the respective concentrations.

#### Mixture II (PAR and DOM)

##### Preparation of stock and working solutions

Standard solutions of PAR and DOM were prepared separately in methanol containing 1000 µg/mL each. Accurate volumes from the previously prepared standard solutions of PAR and DOM were transferred into two separate sets of 10-mL volumetric flasks and diluted to volume with methanol to obtain final PAR and DOM concentration ranges of 3–70 µg/mL and 2.5–15 µg/mL, respectively. The generated working solutions’ zero order absorption spectra were recorded by scanning between 200 and 400 nm against a solvent blank. Excel software was used for treatment of the stored spectra.

##### Application of ratio difference method

To obtain ratio spectra for PAR, the recorded absorption spectra were divided by the spectrum of 50 µg/mL standard DOM solution. By recording the difference between the ratio spectra amplitudes at 256 and 288 nm versus the appropriate PAR concentrations, the regression equation was computed to create PAR calibration curve. The determination of DOM was accomplished by dividing the recorded DOM UV spectra by the spectrum of 50 µg/mL standard PAR as the divisor. By measuring the difference in ratio spectra values at 216 and 288 nm against the respective concentrations, the regression equation was developed in order to produce DOM calibration curve.

### Preparation of sample solutions (obtained from laboratory-made tablets)

Laboratory-made tablets with 325 mg PAR and 7.5 mg MEL [13] for combination I and 500 mg PAR and 20 mg DOM [14] for mixture II were the formulations tested in the study. The pills were prepared using inactive components such as magnesium stearate, colloidal silica (Aerosil), microcrystalline cellulose (Avicel), and maize starch. After grinding of the prepared tablets, a quantity of the lab-made tablets equal to 325 mg PAR and 7.5 mg MEL was weighed for mixture (I). First, 5 mL of DMF were added to dissolve MEL. On the other hand, a quantity equal to 500 mg PAR and 20 mg DOM was weighed for mixture (II). The active components in each mixture powdered sample were extracted with 20 mL of methanol, sonicated for 30 min, and filtered into a 100 mL volumetric flask. The residue was rinsed twice with 25 mL of methanol. Mixture I stock sample solution was completed with methanol to achieve a final concentration of 3250 µg/mL PAR and 75 µg/mL MEL. Solution for mixture II was diluted with methanol to achieve a final concentration of 5000 µg/mL PAR and 200 µg/mL DOM. Aliquots from the stock tablets extracts were further diluted with methanol to reach the specified concentration ranges which were scanned spectrophotometrically and analyzed as the general procedures. Recoveries were calculated using similarly analyzed standard solutions.

## Results and discussion

### Development of Zero-order and First-order derivative spectrophotometric methods for analysis of mixture I

#### Spectral characteristics of the analytes

**Fig. 2:**
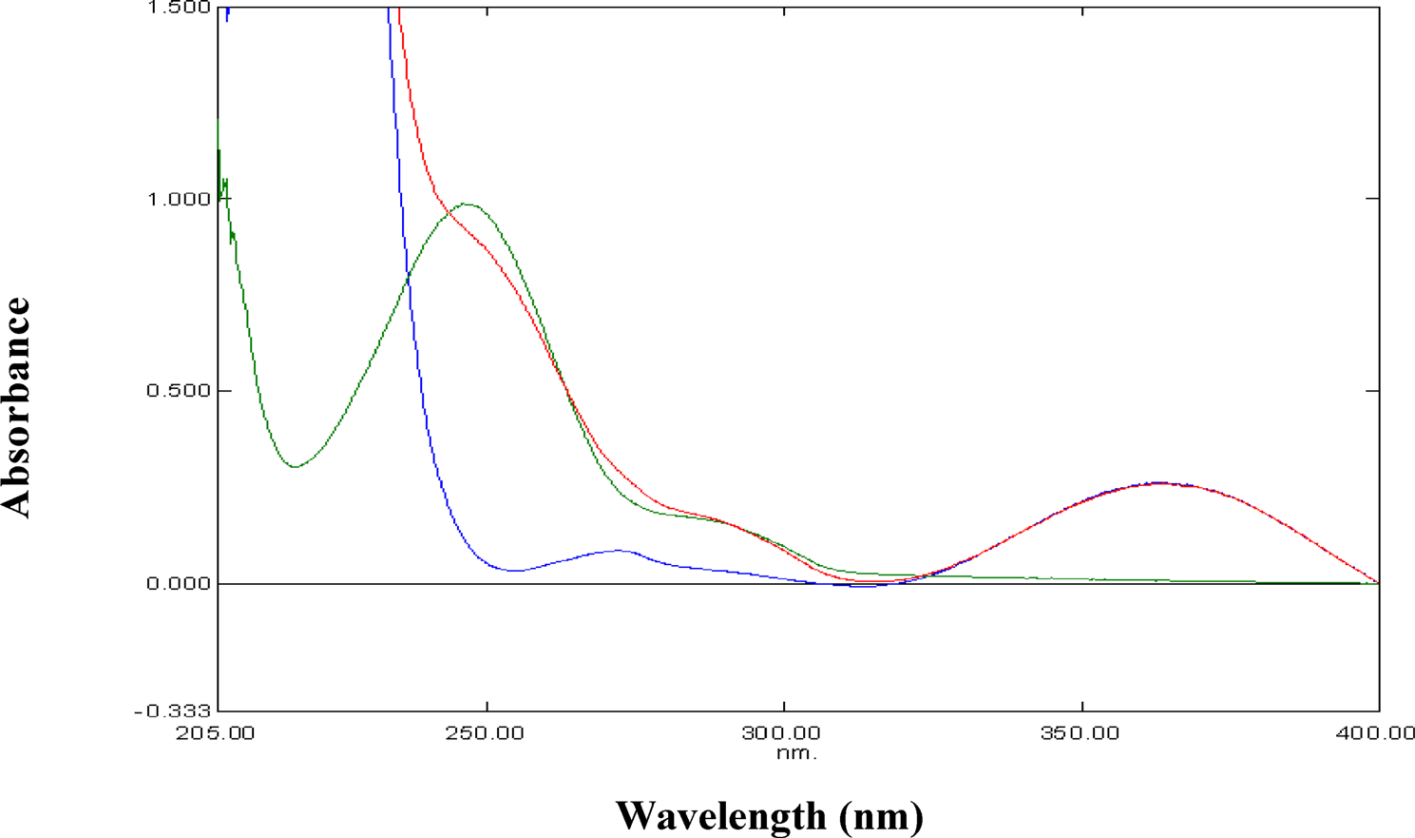
Zero order absorption spectra of 10 µg/mL PAR (), 10 µg/mL MEL () and their mixture () in methanol

**Fig. 3:**
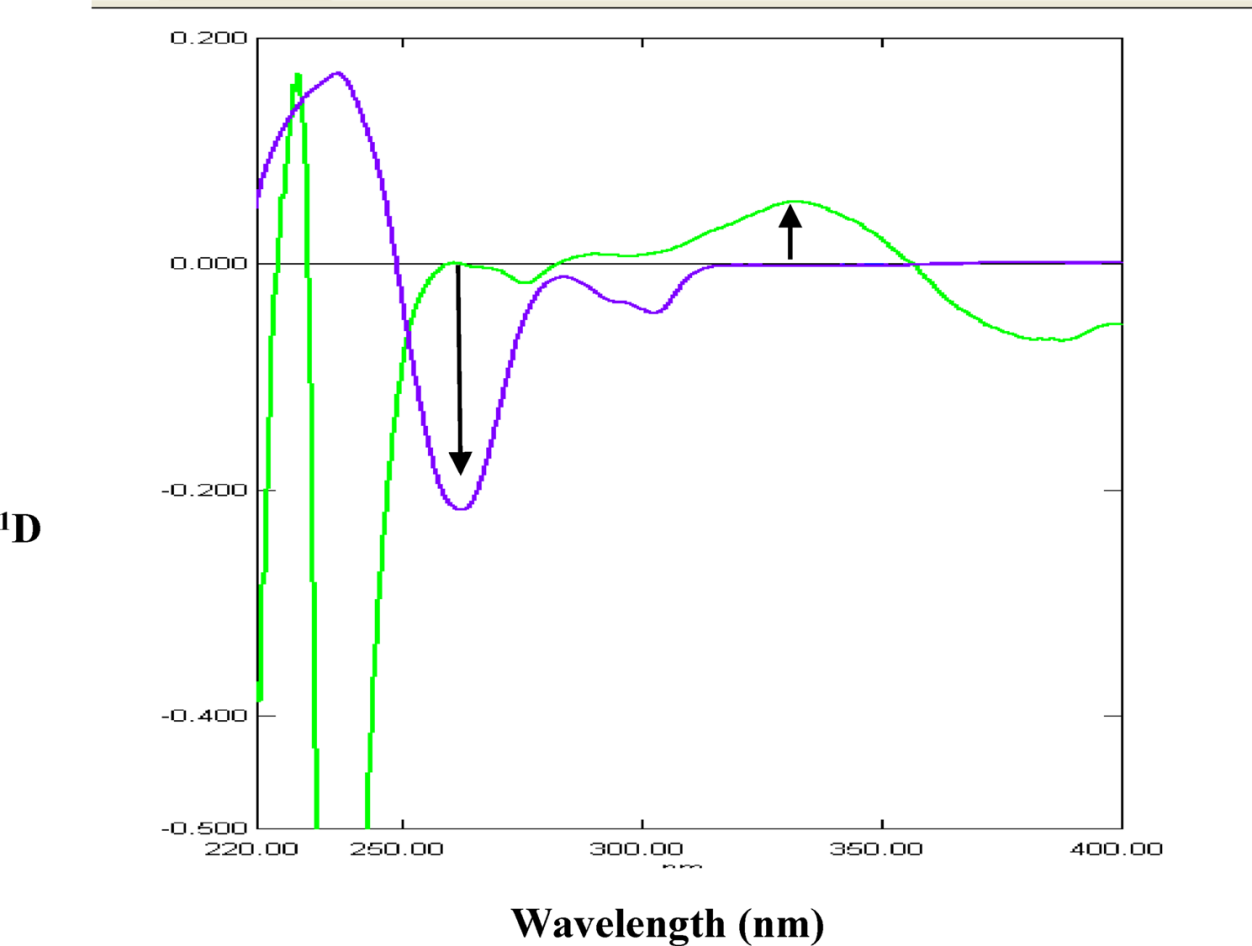
First derivative spectra (^1^D) of 10 µg/mL PAR () and 10 µg/mL MEL () in methanol

Figure 2 represents the UV absorption spectra of PAR, MEL and their mixture. The zero-order absorption spectra of PAR and MEL show overlap in the range 200–320 nm. Fortunately, MEL spectrum reveals a prominent maximum at 361 nm without any contribution from PAR. Accordingly, zero-order spectrophotometry can be applied to the determination of MEL by measuring absorbance at 361 nm in samples containing PAR. This was further refined with the use of first-derivative (^1^D) spectrophotometry by measuring the ^1^D amplitude of MEL at 342 nm where PAR showed no interference at those working wavelengths (Fig. 3). In addition, first-derivative (^1^D) spectrophotometry was advantageous for the determination of PAR in the presence of MEL at its zero-crossing 262 nm without any interference from MEL (Fig. 3).

#### Optimization of the spectrophotometric conditions

The effect of various diluting solvents was examined such as, methanol, distilled water and HCl to determine which solvent would be suitable for the preparation of the working solutions. HCl was one of the suggested solvents as it was reported in some papers to be the solvent of choice for working solution preparation. In view of that, a stability study was performed by using 0.05 M HCl as a solvent for a duration of 2 h with 15 min interval, for both drugs separately. It was found that PAR’s absorbance was fluctuating during the 2 h period of the study. As for MEL, it showed a decrease in absorbance after 1.5 h. Therefore, HCl was excluded as a diluting solvent.

**Fig. 4:**
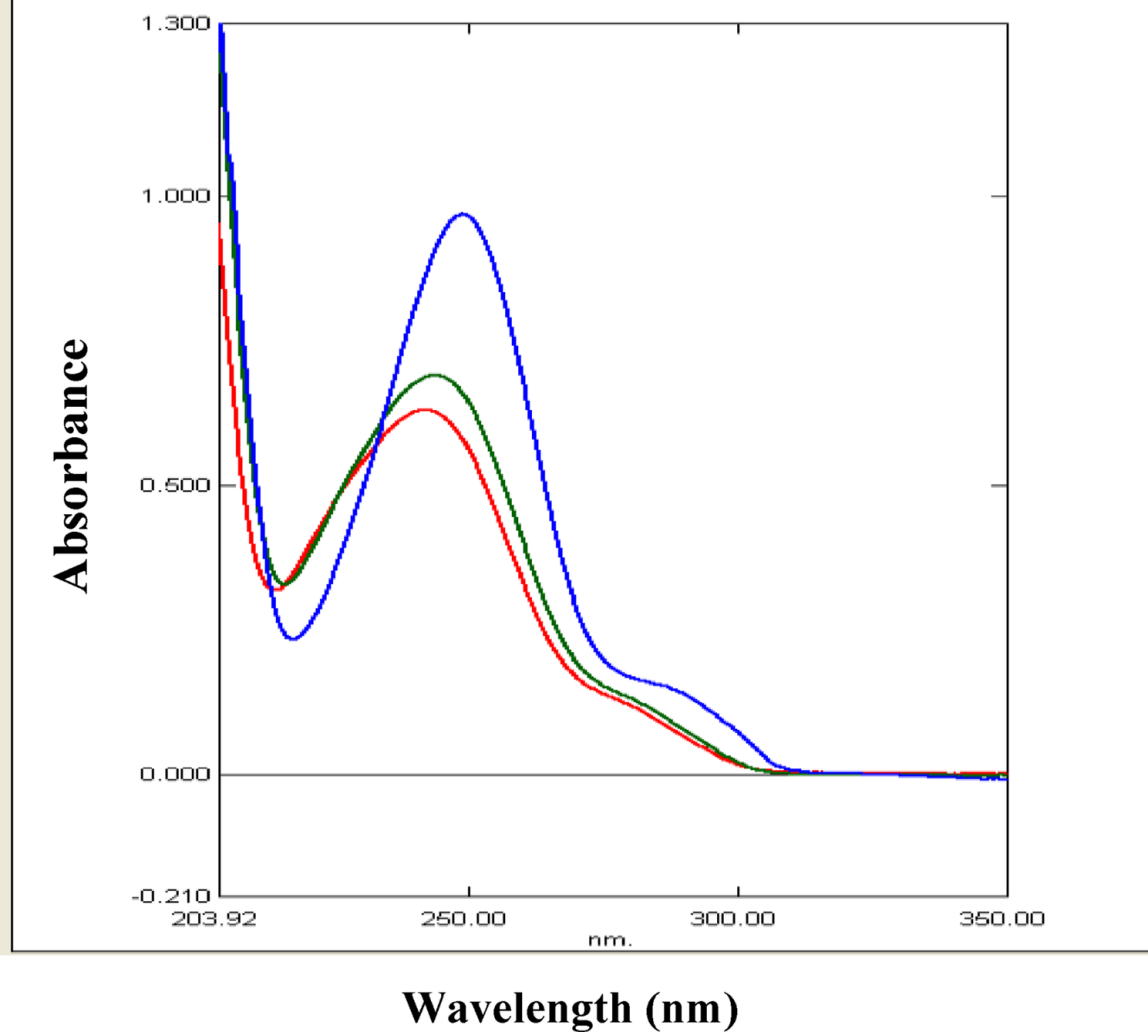
Absorption spectra of 10 µg/mL PAR in different solvents: 0.10 M HCl (), distilled water () and methanol ()

**Fig. 5:**
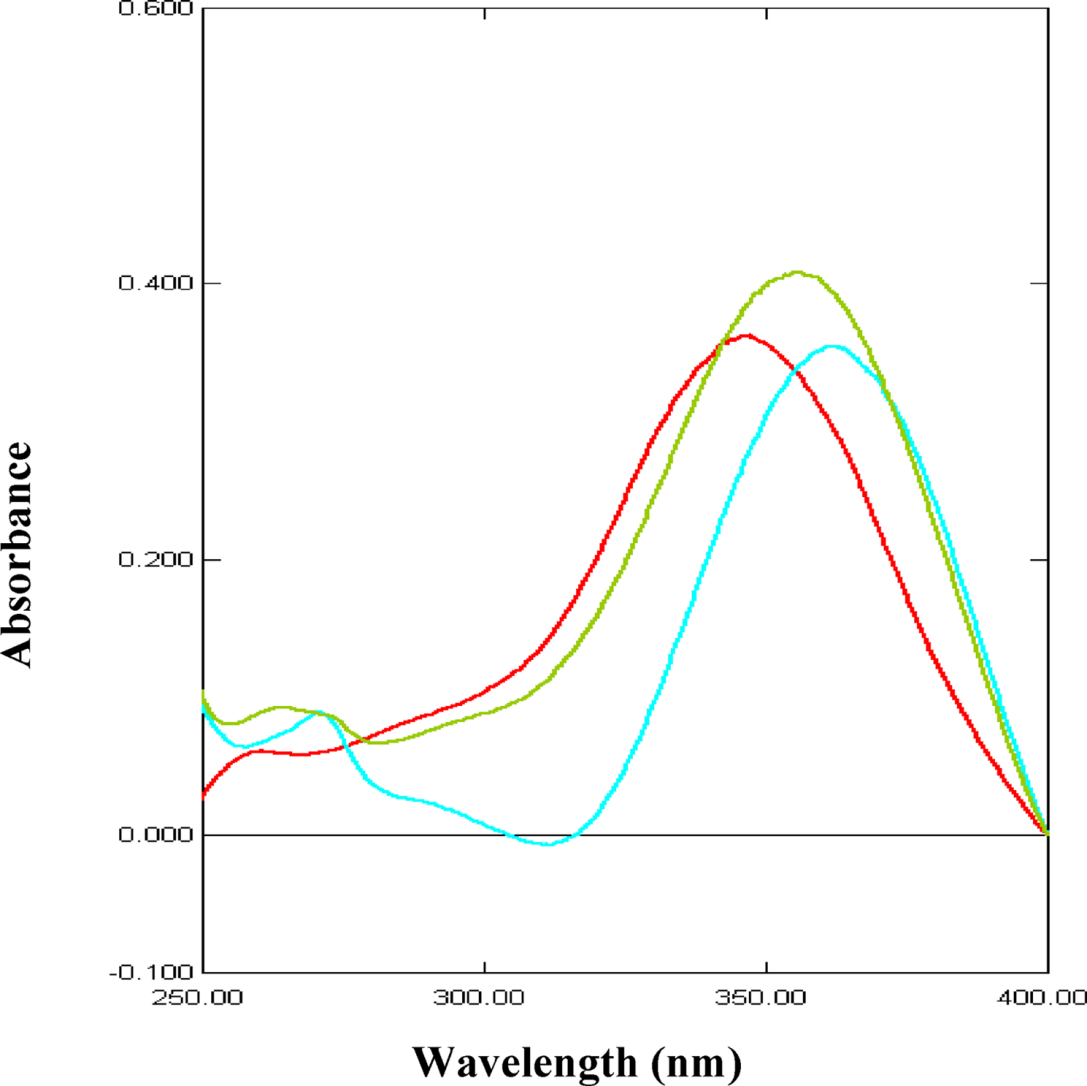
Absorption spectra of 10 µg/mL MEL in different solvents: 0.10 M HCl (), distilled water () and methanol ()

Methanol and distilled water were then tested to determine which one would be the solvent of choice. Finally, methanol showed better absorbance and spectrum shape so it was the solvent of choice for this study, as illustrated in Figs. 4 and 5. Furthermore, wavelength interval (Δλ) was studied for its effect on the first derivative curves. Different Δλ values (2, 4, 8 and 16) were experimented, and no significant changes were noticed, so Δλ = 4 was selected. The scaling factor (SF) was also considered. The first derivative curves were procured using different SF values: 1, 5 and 10. It was found that SF = 5 had the best results, therefore it was the SF of choice.

### Development of the ratio difference spectrophotometric method for analysis of mixture II

**Fig. 6:**
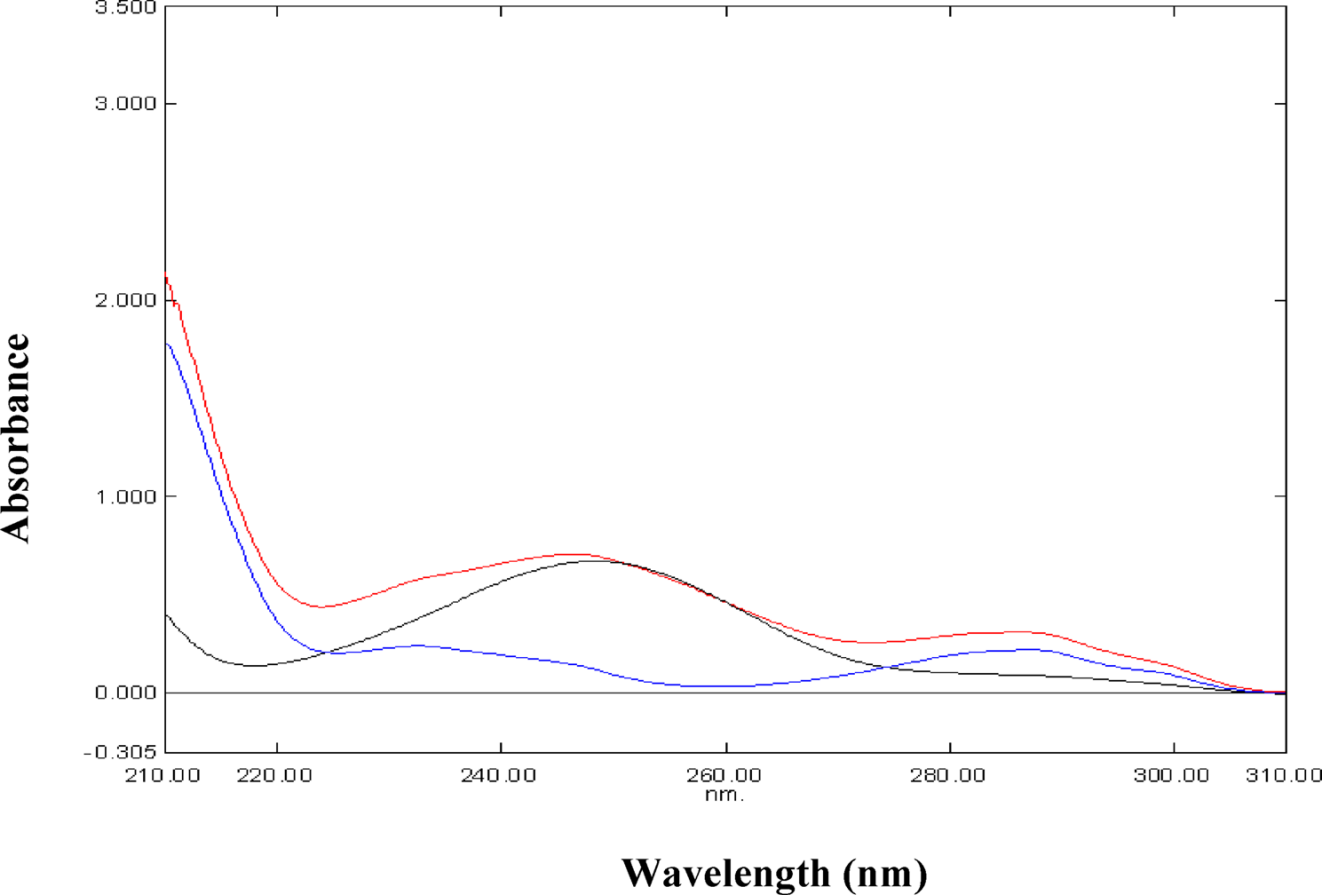
Absorption spectra of 8 µg/mL PAR (), 8 µg/mL DOM () and their mixture () in methanol

The conventional UV spectrophotometric measurement was not suitable for simultaneous determination of PAR and DOM due to their overlapping absorption spectra (Fig. 6). A straightforward one-step correction method based on absorbance ratio spectra was created to get over the mutual interference of each compound in determination of the other. The difference between two points on the ratio spectra of the combination is directly proportional to the concentration of the constituent of interest, regardless of the concentration of the other constituent, according to the fundamental concept of the ratio difference method. For a combination of two medications with overlapping spectra, X and Y, we can assess X via dividing the mixture spectrum by the spectrum of a recognized concentration of Y as a divisor (YꞋ). The division creates a new curve that represents

$$\frac{\text{(X + Y)}}{\text{Y}'} = \frac{\text{X}}{\text{Y}'} + \frac{\text{Y}}{\text{Y}'} = \frac{\text{X}}{\text{Y}'} + \text{constant}$$

To cancel the constant term Y/ YꞋ and subsequently eliminate interference of compound Y, select two wavelengths λ1 and λ2 on the computed ratio spectrum and subtract the amplitudes between these two positions.

**Fig. 7:**
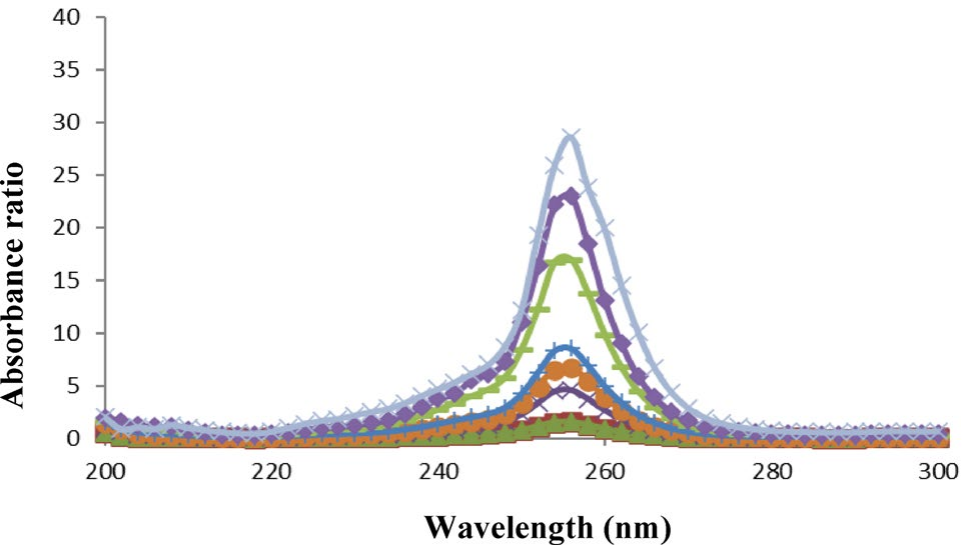
Ratio spectra of 3, 5, 8, 12, 15, 30, 40 and 70 µg/mL PAR using 50 µg/mL DOM as divisor

**Fig. 8:**
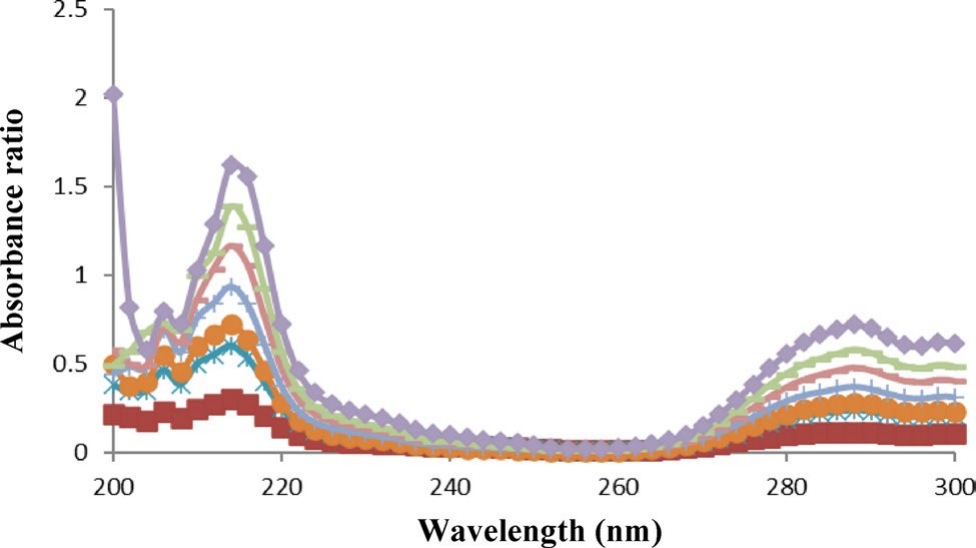
Ratio spectra of 2.5, 5, 6, 8, 10, 12 and 15 µg/mL DOM using 50 µg/mL PAR as divisor

Concerning the proposed method, by dividing the stored scanned spectra of the mixture (amplitude by amplitude) by a standard DOM (50 µg/mL) as a divisor, ratio spectra that indicate PAR/DOM + constant were produced. The constant interfering component was removed by subtracting the amplitudes at 256 and 288 nm. Then, using the created regression equation, PAR concentration can be determined. Similarly, the concentration of DOM in the mixture can be calculated quantitatively by using standard PAR (50 µg/mL) as a divisor, and subtracting the ratio spectra amplitudes at the chosen wavelengths of 216 and 288 nm. The resulting values were then used to calculate concentration of DOM from the developed regression equation. Figures 7 and 8 display ratio spectra of serial concentration levels for standard PAR and DOM, respectively.

The wavelengths used for the determination of both drugs were chosen depending on the best sensitivity obtained, accurate results in analysis of synthetic mixtures in different proportions and reproducibility in measurements. A study was carried out to investigate the impact of the divisor concentration on the calibration graphs. Concentrations of 20, 40 and 50 µg/mL of each drug were inspected for the study of the divisor concentration. Linear equations retrieved upon using these divisor concentrations are given in Table S1 in the Supplementary File. Although the maxima and minima remain at the same wavelengths, the measured amplitudes are proportionally diminished or enhanced depending on whether the divisor’s concentration is increased or decreased. Although the three concentrations generated acceptable linearity parameters, it was found that the 50 µg/mL for both drugs exposed best performance in terms of the highest correlation coefficients, the lowest intercepts and standard deviation of error and the most reproducible results in the analysis of laboratory prepared mixtures.

### Validation of the proposed methods

The developed procedures were validated in accordance with International Council for Harmonization (ICH) standards [15].

#### Linearity and concentration ranges

The proposed methods’ linearity was examined by assessing various concentrations of PAR and MEL (mixture I) and PAR and DOM (mixture II). As per the ICH recommendations, at least five concentrations should be employed. In this linearity testing, no less than seven concentrations were prepared and examined for each analyte. The analysis was conducted under the previously stated experimental settings. The data from the linear equations show high correlation coefficients (*r* ≥ 0.9991) and minimal intercepts, indicating that the calibration graphs are perfectly linear. The linear least squares statistical treatment was used to compute slopes (b), intercepts (a), correlation coefficients (r), and the important standard deviation values S_a_, S_b_ and S_y/x_. Less than 2% made up the RSD% of the slope values (S_b_%), indicating good linearity (Table 1). Moreover, analysis of variance (ANOVA) test can be exploited to corroborate the linear relationship. The major statistical parameter in this test is the F-value which is a ratio retrieved by mean of squares on account of regression divided by the mean of squares caused by residuals. High F values confirm an increase in the regression mean of squares and a decline in the residuals mean of squares. The greater the regression mean of squares; the steeper is the regression line. The smaller the residuals mean of squares; the less is the dispersion of experimental points about the calibration line. Thus, regression lines with high F values (lesser significance F) function much better than those with poor F values. Both the r and F statistical metrics have high values in good regression lines (Table 1).

**Table 1:** Analytical parameters for determination of both mixtures using the proposed spectrophotometric methods

Parameter	Mixture I (PAR – MEL)			Mixture II (PAR – DOM)	
	MEL Zero order	MEL ^1^D	PAR ^1^D	PAR (Ratio difference)	DOM (Ratio difference)
Wavelength (nm)	361	342	262	256 and 288	216 and 288
Concentration range (µg/mL)	3.0–30.0	2.5–30.0	3.0–15.0	3–70	2.5–15
Intercept (a)	– 0.082	– 0.002	0.001	0.040	0.021
S_a_^a^	0.014	3.83 × 10^–4^	0.001	0.149	0.003
Slope (b)	0.053	0.006	0.020	0.543	0.056
S_b_^b^	0.001	2.51 × 10^–5^	6.68 × 10^–5^	0.004	0.0003
RSD% of the slope (S_b_%)	1.887	0.418	0.334	0.737	0.536
Correlation coefficient (r)	0.9991	0.9999	0.9999	0.9999	0.9999
S_y/x_^c^	0.022	0.001	0.001	0.240	0.003
F^d^	3306.89	51235.36	85462.56	15869.32	33297.89
Significance F	1.86 × 10^–9^	5.02 × 10^–13^	8.83 × 10^–8^	1.10 × 10^–6^	9.38 × 10^–11^
LOD^e^ (µg/mL)	0.872	0.210	0.165	0.906	0.177
LOQ^f^ (µg/mL)	2.642	0.638	0.500	2.744	0.536

^a^ Standard deviation of the intercept

^b^ Standard deviation of the slope

^c^ Standard deviation of residuals

^d^ Variance ratio, equals the mean of squares due to regression divided by the mean of squares about regression (due to residuals)

^e^ Limit of detection

^f^ Limit of quantitation

Figures S1 and S2 in the Supplementary File illustrate the calibration graphs of intrinsic absorbance and ^1^D values versus the corresponding concentrations of MEL respectively, while Figure S3 demonstrates the calibration graph of ^1^D data of PAR against its concentrations. Similarly for mixture II, Figures S4 and S5 in the Supplementary File expose the calibration graphs for PAR and DOM respectively. Vertical variations between experimental data points and the least-squares line are shown in the residuals plots in Figures S6, S7 and S8 (for mixture I) and Figures S9 and S10 (for mixture II) [see the Supplementary File] indicating that the obtained data fit with their regression lines.

#### Detection and quantitation limits

The LOQ and LOD were calculated in accordance with the ICH recommendations. LOQ was defined as 10 S_a_ / b and LOD as 3.3 S_a_ / b, where S_a_ is the standard deviation of the calibration curve’s intercept and b is the slope. The calculated values are presented in Table 1.

#### Accuracy and precision

Three concentration levels of PAR and MEL (for mixture I) as well as PAR and DOM (for mixture II) within their linearity ranges were used, with three replicate measurements for each concentration, to study the accuracy of the suggested spectrophotometric platforms (Table 2). Furthermore, the study of the accuracy was extended to the analysis of the examined drugs in laboratory-prepared mixtures in different ratios (Tables 3 and 4) and in laboratory-made tablets (Table 5). The excellent accuracy of the suggested procedures is indicated by the good recoveries achieved and the low percentage error (Er%) values that never exceeded 2%.

**Table 2:** Precision and accuracy for the determination of MEL and PAR (mixture I) and PAR and DOM (mixture II) using the proposed spectrophotometric methods

	Analyte and Method	Nominal value (µg/mL)	Within-day			Between-days		
			Found ± SD^*^ (µg/mL)	RSD (%)	E_r_ (%)	Found ± SD^*^ (µg/mL)	RSD (%)	E_r_ (%)
Mixture I	MEL Zero order (361 nm)	5 12 20	4.99 ± 0.08 12.06 ± 0.21 20.09 ± 0.03	1.60 1.74 0.15	–0.20 0.50 0.45	4.92 ± 0.05 12.21 ± 0.24 20.18 ± 0.20	1.02 1.97 0.99	–1.60 1.75 0.90
	MEL ^1^D (342 nm)	7.5 12 30	7.57 ± 0.10 11.86 ± 0.10 30.12 ± 0.12	1.32 0.84 0.40	0.93 –1.17 0.40	7.46 ± 0.10 11.86 ± 0.10 29.92 ± 0.20	1.34 0.84 0.67	–0.53 –1.17 –0.27
	PAR ^1^D (262 nm)	3 5 15	2.99 ± 0.06 4.99 ± 0.04 14.90 ± 0.14	2.01 0.80 0.94	–0.33 –0.20 –0.67	2.99 ± 0.03 5.01 ± 0.05 14.92 ± 0.11	1.00 1.00 0.74	–0.33 0.20 –0.53
Mixture II	PAR (Ratio difference)	8 15 60	8.04 ± 0.01 15.20 ± 0.02 60.34 ± 0.82	0.12 0.13 1.36	0.50 1.33 0.57	7.85 ± 0.16 15.30 ± 0.14 59.76 ± 0.89	2.04 0.92 1.49	–1.88 2.00 −0.40
	DOM (Ratio difference)	5 10 15	4.92 ± 0.10 9.90 ± 0.10 14.77 ± 0.18	2.03 1.01 1.22	–1.60 –1.00 –1.53	4.93 ± 0.07 9.96 ± 0.13 14.87 ± 0.13	1.42 1.31 0.87	–1.40 –0.40 –0.87

* Mean ± standard deviation for three determinations

**Table 3:** Analysis of laboratory-prepared mixtures of PAR and MEL (mixture I) using the proposed spectrophotometric methods

Nominal value (µg/mL)		Found ± SD^a^ (µg/mL)			RSD(%)^b^			Er(%)^c^		
MEL	PAR	MEL Zero order (361 nm)	MEL ^1^D (342 nm)	PAR ^1^D (262 nm)	MEL Zero order (361 nm)	MEL ^1^D (342 nm)	PAR ^1^D (262 nm)	MEL Zero order (361 nm)	MEL ^1^D (342 nm)	PAR ^1^D (262 nm)
10	10	9.95 ± 0.02	10.16 ± 0.14	9.98 ± 0.06	0.20	1.38	0.60	– 0.50	1.60	– 0.20
5	10	5.03 ± 0.06	5.03 ± 0.05	9.98 ± 0.11	1.19	0.99	1.10	0.60	0.60	– 0.20
10	5	9.92 ± 0.04	10.17 ± 0.12	5.07 ± 0.03	0.40	1.18	0.59	– 0.80	1.70	1.40
2.5	12.5	2.51 ± 0.04	2.51 ± 0.05	12.28 ± 0.03	1.59	1.99	0.24	0.40	0.40	– 1.76
0.3	13	–	–	13.08 ± 0.03	–		0.23	–	–	0.62
3	130	3.04 ± 0.04	3.04 ± 0.06	–	1.32	1.97	–	1.33	1.33	–

^a^ Mean ± standard deviation for three determinations

^b^ % Relative standard deviation

^c^ % Relative error

**Table 4:** Determination of PAR and DOM (mixture II) laboratory-prepared mixtures using the proposed spectrophotometric method

Nominal value (µg/mL)		Found ± SD^a^ (µg/mL)		RSD(%)^b^		E_*r*_(%)^c^	
PAR	DOM	PAR	DOM	PAR	DOM	PAR	DOM
8	8	7.85 ± 0.01	8.14 ± 0.04	0.13	0.49	– 1.88	1.75
16	8	15.73 ± 0.09	7.89 ± 0.05	0.57	0.63	– 1.69	– 1.38
5	10	4.98 ± 0.03	10.02 ± 0.03	0.60	0.30	– 0.40	0.20
50	5	49.50 ± 0.96	4.99 ± 0.02	1.94	0.40	– 1.00	– 0.20
62.5	2.5	62.14 ± 0.40	2.49 ± 0.01	0.64	0.40	– 0.58	– 0.40

^a^ Mean ± standard deviation for five determinations

^b^ % Relative standard deviation

^c^ % Relative error

**Table 5:** Application of the proposed spectrophotometric methods for the analysis of PAR-MEL (mixture I) and PAR-DOM (mixture II) in laboratory-made tablets

Parameters	Mixture I					Mixture II			
	Proposed methods			Reported method [3]		Proposed method		Reported method [7]	
	MEL Zero order (361 nm)	MEL ^1^D (342 nm)	PAR ^1^D (262 nm)	PAR	MEL	PAR	DOM	PAR	DOM
%Recovery ± SD^**a**^	99.43 ± 0.85	99.61 ± 0.96	100.55 ± 0.85	100.14 ± 0.54	99.86 ± 0.34	98.67 ± 0.64	101.97 ± 0.50	98.50 ± 0.52	101.53 ± 1.27
RSD%^**b**^	0.85	0.96	0.85	0.54	0.34	0.65	0.49	0.53	1.25
t	0.94	0.50	0.82			0.35	0.55		
F	6.30	8.04	2.51			1.53	6.33		

^a^ Mean ± standard deviation for four determinations and three determinations for mixture I and II, respectively

^b^ % Relative standard deviation

Theoretical values for t and F at *P* = 0.05 are 2.45 and 9.28, respectively (mixture I) and 2.77 and 19.00, respectively (mixture II)

For assessment of method repeatability, the relative standard deviations of three intra-day (within-day) analyses for PAR and MEL (mixture I) as well as PAR and DOM (mixture II) were calculated (Table 2). Regarding the intermediate precision, the three selected drug concentrations were examined in triplicate over three separate days (between-day analysis) using the suggested procedures. In both types of analysis, the percentage relative SD (RSD%) results did not exceed 2%, demonstrating the agreeable precision of the developed procedures. (Table 2).

#### Selectivity

Through the preparation of various mixtures of PAR and MEL (mixture I) as well as PAR and DOM (mixture II) within the previously stated linearity ranges, the selectivity of the developed methods was assessed. PAR to MEL ratios included 1:1, 2:1, 1:2 and 5:1 whereas, PAR to DOM ratios comprised 1:1, 2:1, 1:2 and 10:1. Additionally, ratios of PAR/ MEL 130:3 and PAR/DOM 25:1 which resemble the real ratios in commercial tablet dosage forms were examined. Satisfactory recovered concentrations, RSD % and Er % values were achieved by utilizing the zero-order measurements at 361 nm for MEL and first-order derivative (^1^D) at 262 nm and 341 nm for PAR and MEL respectively (Table 3), and the ratio difference method for PAR and DOM mixture (Table 4). These findings support the convincing selectivity of the developed methods for the simultaneous assessment of these pharamaceuticals in various ratios mixtures.

#### Stability of solutions

In order to ensure that no appreciable spectrophotometric changes occurred, the stability of the standard and working solutions in methanol were checked for at least 6 h, and no significant change was observed. The stock solutions of PAR and DOM maintained their stability for at least one week in the refrigerator at 4 °C. However, MEL stock solution should be freshly prepared.

### Assay of laboratory-made tablets

PAR and MEL determination in mixture I as well as PAR and DOM (mixture II) in their laboratory-made tablets was achieved using the developed spectrophotometric approaches; due to a scarcity of their commercial pharmaceutical formulations in the local market. The results of the analysis showed good accuracy and precision as evidenced by % recovery, SD, and RSD% values as illustrated in Table 5. The obtained results were statistically compared to those of a spectrophotometric simultaneous equations reference method [3] for mixture I and first derivative spectrophotometry [7] for mixture II using the t-test and variance ratio F-test. The calculated values did not exceed the critical ones, indicating no significant difference between the devised and available published methods (Table 5). Also, these findings guarantee the absence of influence from the co-formulated tablet additives.

## Evaluation of greenness and comparison with some reported methods

Analytical processes should be evaluated for their environmental impact. The use of several greenness evaluation tools is advocated in order to provide a more significant comparison and logical ranking of different analytical methodologies based on their eco-friendliness attributes.The Analytical Eco-Scale [16] and the novel Analytical Greenness metric (AGREE) [17] were the two approaches employed in this study to assess the degree of greenness. Several recent pharmaceutical analysis studies have used such tools to detect levels of greenness [18–23].

The analytical Eco-Scale is based on the concept that green assessment must have a score of 100 to be considered perfect. If the analytical technique deviates from the ideal green analysis, penalty points are assigned to each of the parameters (amount of reagents, hazards, energy, and waste). The following formula should be used to calculate the final score, taking into account the total number of penalty points for the entire procedure:

$$ {{Analytical Eco - Scale = 100 - total penalty points}}. $$

A scale with the following values is used to rank the calculation’s outcome: >75 represents excellent green analysis, >50 represents acceptable green analysis, < 50 represents inadequate green analysis [16].

The spectrophotometric methods introduced in this work for the analysis of both combinations have great green performance, with few detrimental consequences on the environment or human health. This was demonstrated for mixture I through the calculated analytical Eco-Scale score of 91 which proved better score than the HPTLC [6] method having score of 76. However, the proposed method exhibited almost equal green preformance compared to the reported HPLC [1] and spectrophotometric methods [3] that presented Eco-scale scores of 90 and 96, respectively (Table 6). Similarly for mixture II, the proposed ratio-difference method was well-thought-out to have excellent green performance according to its computed analytical Eco-Scale score of 91 (Table 6), which is nearly equal to that of the reported spectrophotometric methods [7, 8] revealing scores of 91 and 97. Finally, the reported HPLC [9] and HPTLC [11] methods showed lower scores of 86 and 82 respectively.

**Table 6:** Penalty points of the proposed and the reported methods according to the analytical Eco-scale

Mixture I				
Parameter	Proposed Spectrophotometric Methods (zero & first order derivative)	Reported Spectrophotometric Methods [3] (Simultaneous equations & Absorbance ratio methods)	Reported HPLC Method [1]	Reported HPTLC Method [6]
Methanol	6	**-**	6	6
0.1 N NaOH	–	1	–	–
Phosphate Buffer	–	–	0	–
Toluene	–	–	–	6
Ethyl acetate	–	–	–	4
Formic acid	–	–	–	6
Energy	0	0	1	1
Occupational hazards	0	0	0	0
Waste	3	3	3	1
PPs	9	4	10	24
Eco-scale score	91	96	90	76

Parameter	Proposed Spectrophotometric Method	Reported Spectrophotometric Method [8]	Reported HPLC Method [9]	Reported HPTLC Method [11]	Reported Spectrophotometric Method [7]
Methanol	6	**-**	6	6	6
Distilled water	–	0	–	–	–
Acetonitrile	–	–	4	–	–
Acetone	–	–	–	4	–
Carbon tetrachloride	–	–	–	4	–
Energy	0	0	1	1	0
Occupational hazards	0	0	0	0	0
Waste	3	3	3	3	3
PPs	9	3	14	18	9
Eco-scale score	91	97	86	82	91

Further evaluation of the procedures’ greenness was conducted using the open-source, inclusive and informative AGREE tool. This tool has the advantage of considering the 12 guiding principles of green analytical chemistry (GAC) within its software. The free software makes it feasible for the straightforward evaluation process. A common scale with a 0–1 range is created from each of the 12 input variables. The sum of the assessment outcomes for the 12 principles produces the final assessment result. The overall score (number close to 1) is displayed in the middle of the pictogram, with a (red to green) color denoting the inclusive assessment process. The color in the segment with each criterion’s matching number represents how well the procedure performed in each of the assessment criteria.

Consequently, the greenness was intensively assessed, and an overarching comparison between the established spectrophotometric methods and the documented chromatographic and spectrophotometric methods was developed (Table 7). The AGREE tool showed that when compared to other reported spectrophotometric approaches [3], the proposed method for mixture I revealed nearly equal AGREE analytical score of 0.82. Both the reported HPLC-DAD method [1] and HPTLC method [6] exposed lower AGREE scores of 0.79 and 0.80, respectively. This may be attributed to the fact that the energy used in chromatographic techniques is higher than that for spectrophotometric methods. Furthermore, the HPTLC approach used organic solvents as the mobile phase, including toluene, methanol, ethyl acetate and formic acid, whereas the suggested method used only methanol. Similarly for mixture II, AGREE tool showed that the proposed method displayed nearly comparable AGREE analytical score of 0.82 relative to other available spectrophotometric methods [7, 8]. In contrast, the published HPLC [9] and the HPTLC [11] methods had lower counts of 0.76 and 0.78, respectively. This is because the mobile phase in the previous techniques comprised hazardous solvents such as methanol and acetonitrile for the HPLC, and methanol, acetone and carbon tetrachloride for the HPTLC method. Using the two metrics together, we were able to deduce that the recoemmneded spectrophotometric techniques pose no threat to the environment or human health and are of superior or analogous greenness to other documented methods.

**Table 7:** Greenness assessment and comparison of the proposed and reported methods using AGREE tool

Mixture I					
Proposed Spectrophotometric Methods (zero & first order derivative)	Reported Spectrophotometric Methods [3] (Simultaneous equations & Absorbance ratio methods)	Reported HPLC Method [1]	Reported HPTLC Method [6]		
			

Mixture II				
Proposed spectrophotometric method	Reported spectrophotometric method [8]	Reported HPLC Method [9]	Reported HPTLC Method [11]	Reported Spectrophotometric Method [7]
				

## Conclusion

In this study, simple, selective and reliable spectrophotometric methods for the simultaneous determination of PAR and MEL (mixture I) along with PAR and DOM (mixture II) in bulk form and in laboratory-made tablets were described. The developed methods are based on the simple zero and first-order derivative measurements in mixture I, whereas the drugs of mixture II were assayed using ratio difference method. Compared to previously published works, the proposed methods offer the following advantages: low cost, rapid measurements, simplicity, and low solvent use. The reliability of the proposed methods for simultaneous analysis of these binary mixtures in laboratory-made tablets is demonstrated by the statistical analysis of the obtained results. In order to assess the greenness of the proposed spectrophotometric methods, two distinct metrics were utilized (Analytical Eco-Scale and AGREE). The greenness were compared to some other reported methods. The described approaches were found to be as green as the reported methods. Therefore, the proposed methodologies are suitable for regular PAR and MEL analysis, as well as PAR and DOM analysis in combination dosage forms. They are additionally useful in monitoring the quality of bulk manufacture of the provided pharmaceuticals.

## Supplementary Information

Below is the link to the electronic supplementary material.

Supplementary Material 1.

## Data Availability

All of the data analyzed in this study is included in the manuscript and supplementary file.
