# Supplementary material for: Simple spectrophotometric methods for the quantitative analysis of two binary mixtures containing paracetamol as a major component
Source: BMC Chem. 2025 Oct 27;19(1):284. doi: 10.1186/s13065-025-01643-7 (PMC12560461; doi:10.1186/s13065-025-01643-7)
Supplement: Supplementary file 1 — Supplementary Material 1. [file 13065_2025_1643_MOESM1_ESM.pdf]

Supplementary Material

Simple Spectrophotometric Methods for the Quantitative Analysis of Two Binary Mixtures Containing Paracetamol as a Major Component

Karin M. Guirguis, May M. Zeid, Rasha A. Shaalan and Tarek S. Belal

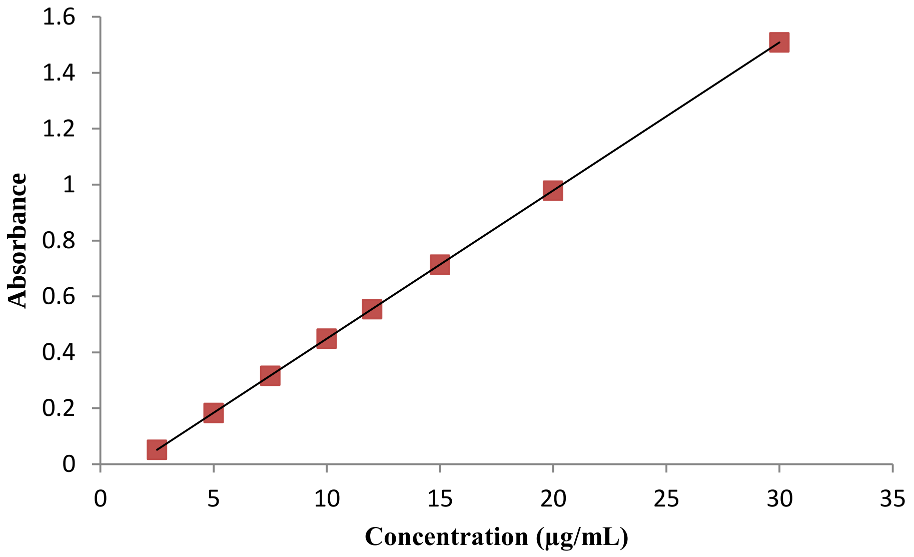

Figure S1: Calibration graph for the determination of MEL in methanol using zero-order method at 361 nm.

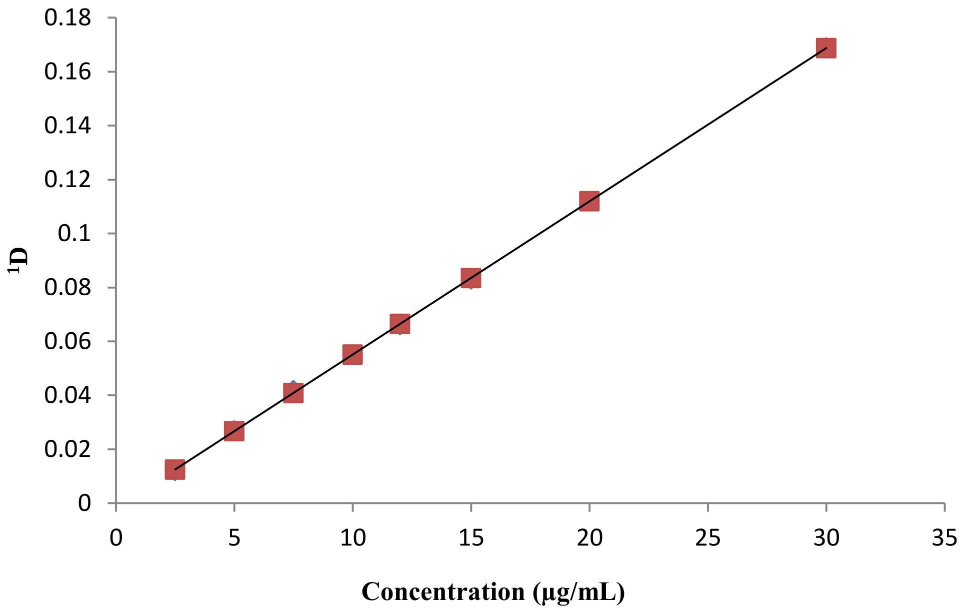

Figure S2: Calibration graph for the determination of MEL in methanol using first-order derivative (^1D) method at 342 nm.

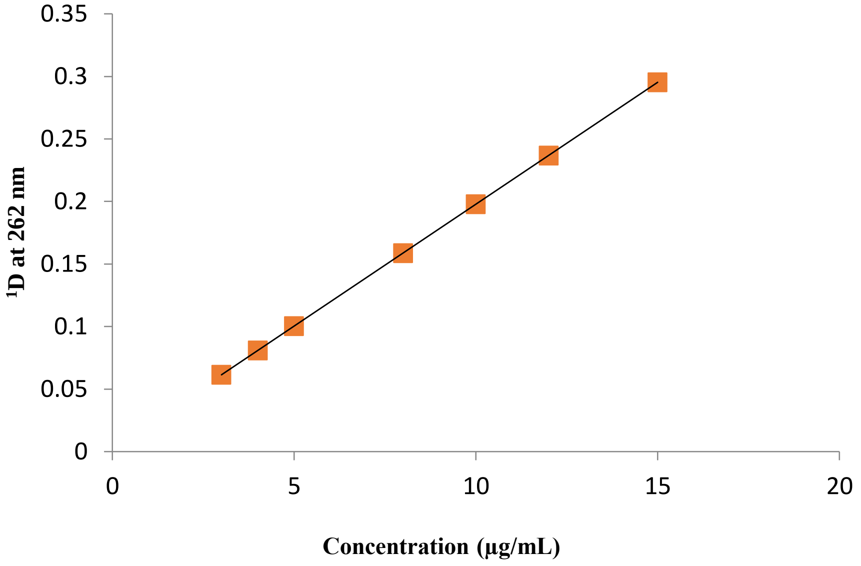

Figure S3: Calibration graph for the determination of PAR in methanol using first-order derivative (^1D) method at 262 nm.

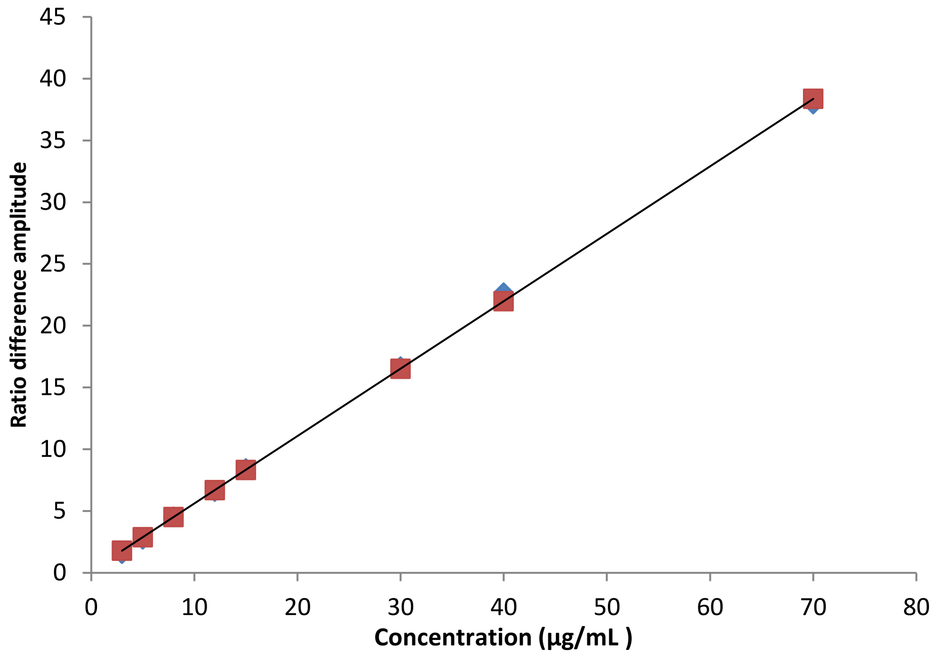

Figure S4: Calibration graph for the determination of PAR using the ratio difference method.

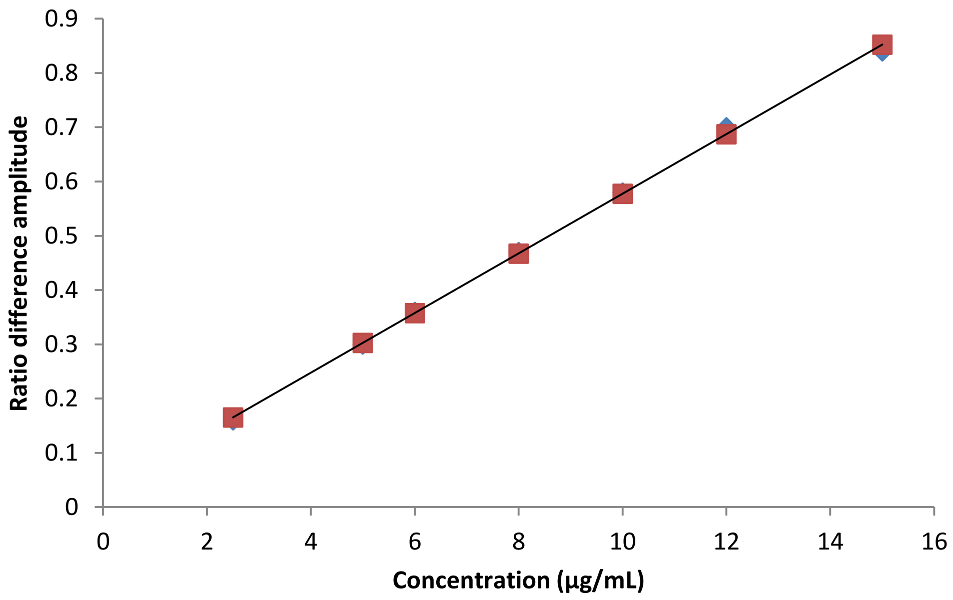

Figure S5: Calibration graph for the determination of DOM using the ratio difference method.

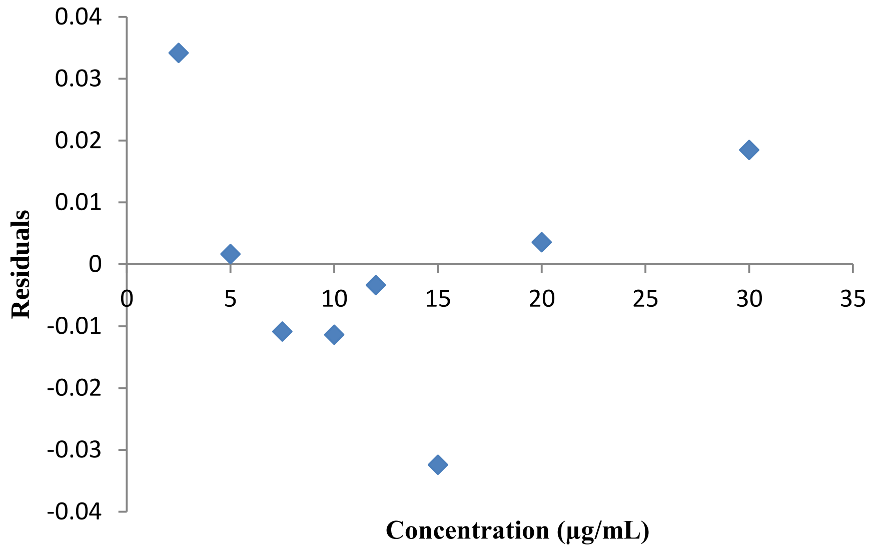

Figure S6: Residuals plot of the regression data for determination of MEL using zero-order method at 361 nm.

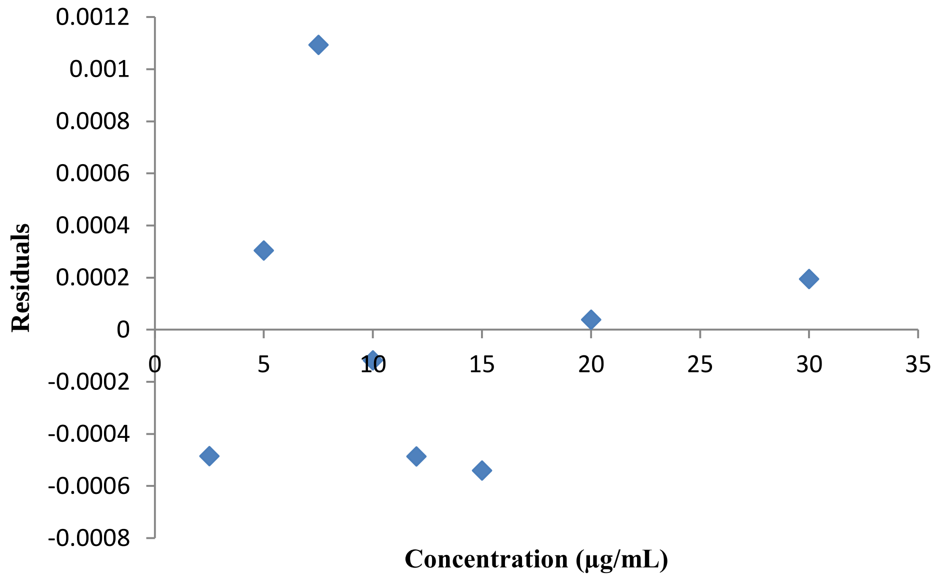

Figure S7: Residuals plot of the regression data for determination of MEL using ^{1}D method at 342 nm.

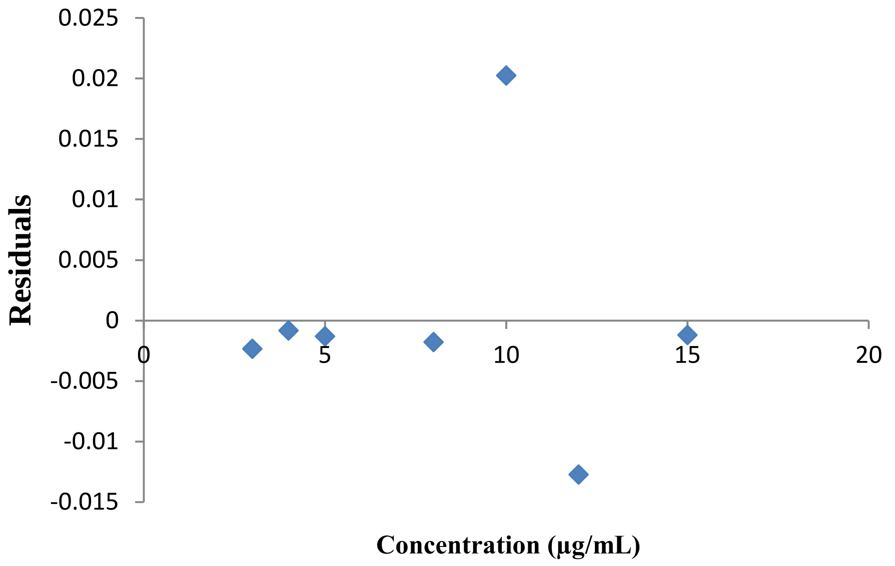

Figure S8: Residuals plot of the regression data for determination of PAR using [1]D method at 262 nm.

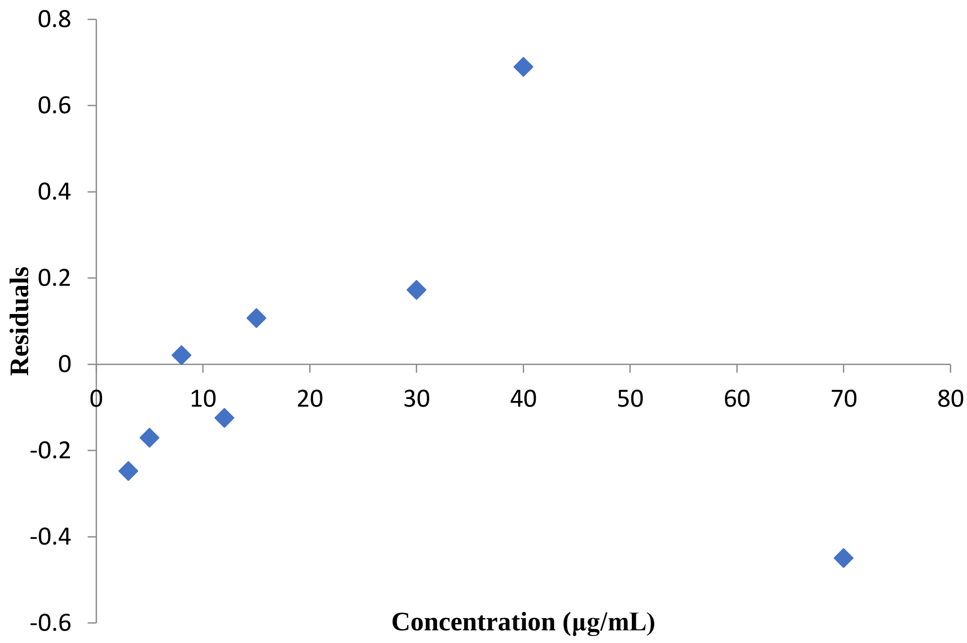

Figure S9: Residuals plot of the regression data for determination of PAR using the ratio difference method.

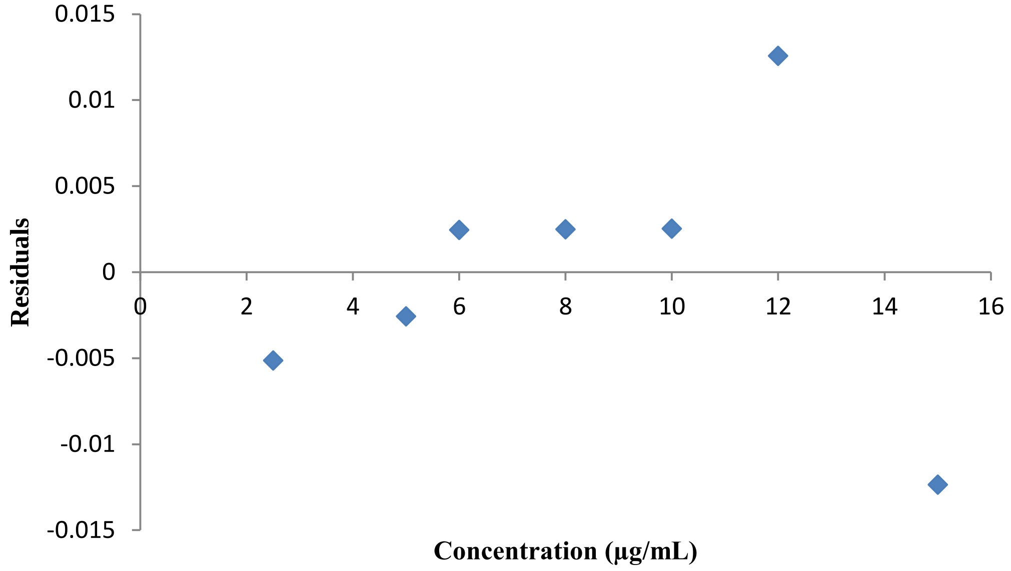

Figure S10: Residuals plot of the regression data for determination of DOM using the ratio difference method.

Table S1: Effect of divisor concentration on determination of PAR-DOM binary mixture using the proposed ratio-difference method.

Analyte and Selected λ (nm)	Divisor conc. (µg/mL)	Intercept ± standard deviation a ± Sa	Slope ± standard deviation b ± Sb	Correlation coefficient (r)
PAR<br>256 and 288	DOM			
	20	0.697 ± 0.398	1.251 ± 0.013	0.9996
	40	0.408 ± 0.275	0.625 ± 0.009	0.9990
	50	0.128 ± 0.192	0.546 ± 0.006	0.9996
DOM<br>216 and 288	PAR			
	20	0.064 ± 0.021	0.124 ± 0.002	0.9991
	40	0.034 ± 0.011	0.069 ± 0.001	0.9992
	50	0.021 ± 0.003	0.056 ± 0.0003	0.9999
